# Five things the intensivist cannot forget in the management of septic shock

**DOI:** 10.62675/2965-2774.20260360

**Published:** 2026-07-22

**Authors:** Carla Daniele Nascimento Pontes, Elisa Estenssoro, Flávia Ribeiro Machado

**Affiliations:** 1 Universidade Federal de São Paulo Escola Paulista de Medicina Hospital São Paulo São Paulo SP Brazil Intensive Care Department, Hospital São Paulo, Escola Paulista de Medicina, Universidade Federal de São Paulo - São Paulo (SP), Brazil; 2 Hospital Interzonal de Agudos General San Martin de La Plata La Plata Buenos Aires Argentina Hospital Interzonal de Agudos General San Martin de La Plata - La Plata, Buenos Aires, Argentina

## INTRODUCTION

Sepsis is a major global health burden and remains one of the leading causes of mortality worldwide, accounting for more than 11 million deaths annually, the majority in low-resource settings.^(1)^ Infections may progress to sepsis, and critically ill patients should be promptly admitted to intensive care units (ICUs). In middle-income countries, the prevalence and mortality of sepsis in ICUs remain unacceptably high.^(2)^ Effective management of septic shock is therefore lifesaving.

**Figure 1:**
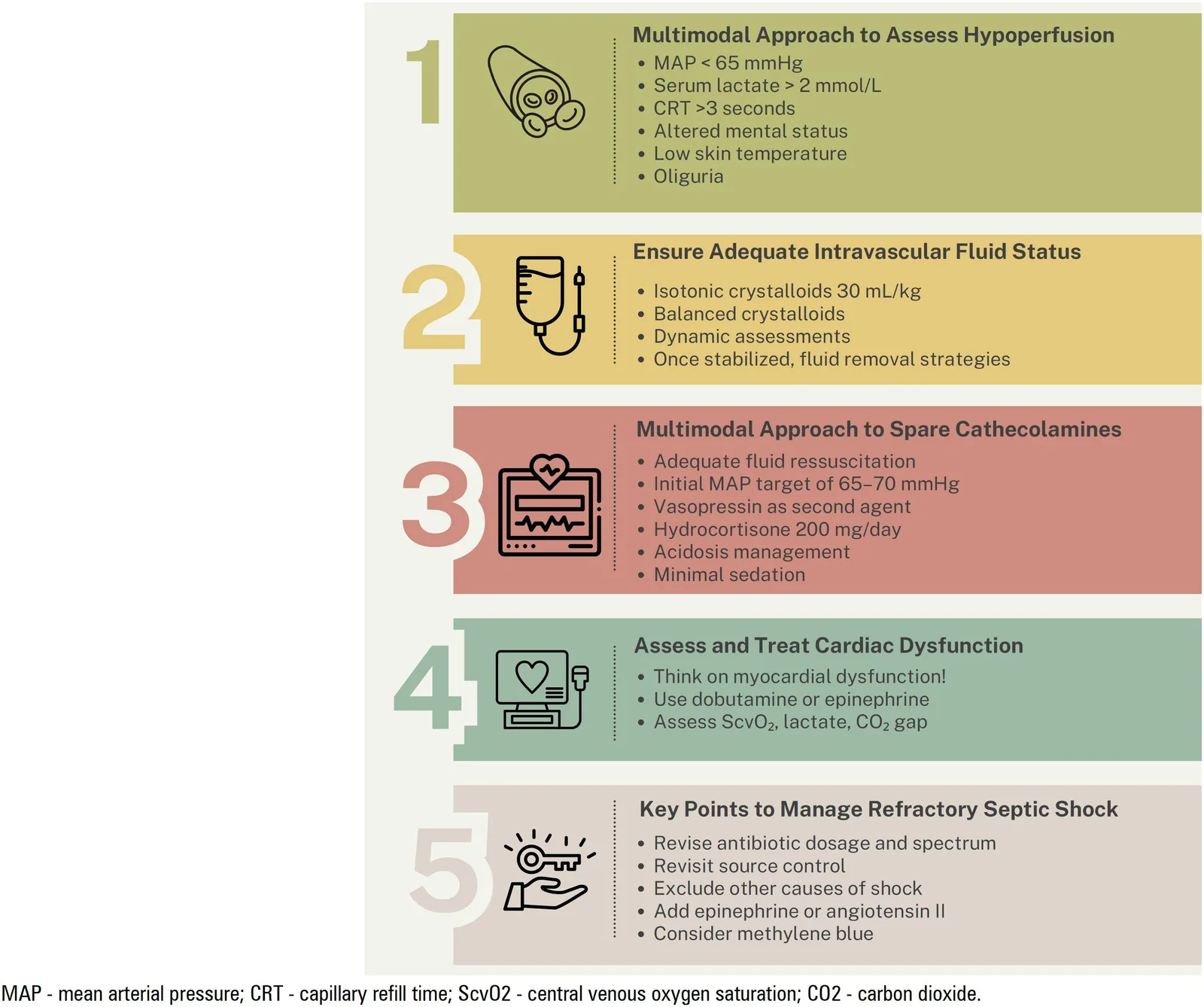
Five things the intensivist cannot forget in the management of septic shock.

For intensivists, septic shock represents a daily challenge, given its high prevalence, associated morbidity and mortality, and the potential for appropriate treatment to improve outcomes. Optimal treatment includes identification of the infectious source, prompt collection of cultures, and timely administration of antimicrobials within the first hour. Source control remains a critical determinant of survival. In this editorial, we outline five key considerations to support clinicians in tailoring hemodynamic management of septic shock (Figure 1).

Figure created by the authors with the assistance of the Canvas Artificial Intelligence tool.

## 1. DO NOT FORGET TO USE A MULTIMODAL APPROACH TO ASSESS HYPOPERFUSION

Circulatory failure in septic shock is multifactorial, involving vasodilation, impaired venous return, ventricular dysfunction, and microvascular abnormalities. Monitoring tissue hypoperfusion is essential, as organ dysfunction may progress rapidly.^(3)^ Microcirculatory hypoperfusion may persist even when blood pressure appears adequate and should therefore be actively sought. In those with hypotension, mean arterial pressure (MAP) should be promptly restored to ≥ 65mmHg, and profound vasoplegia (diastolic arterial pressure < 40mmHg), reflecting loss of vascular tone and jeopardizing coronary perfusion, should be recognized and corrected. Elevated lactate (> 2mmol/L) is a marker of hypoperfusion but is not specific, nonetheless, initial hyperlactatemia remains a validated alarm signal and should trigger urgent assessment. A recent randomized controlled trial demonstrated that prolonged capillary refill time (CRT > 3 seconds) can be used to guide resuscitation.^(4)^ Other clinical indicators include mottling, low skin temperature and oliguria. Hypoperfusion can lead to altered mental status, although sedation and underlying neurologic disease may limit interpretation. Tachycardia and low central venous pressure are nonspecific. A broad, multimodal assessment incorporating these parameters in the clinical context is the most reliable strategy.

## 2. DO NOT FORGET TO ENSURE ADEQUATE INTRAVASCULAR FLUID STATUS

Fluid resuscitation requires attention to both type and amount, balancing the risks of under- and over-resuscitation. International guidelines typically suggest an initial bolus of 30mL/kg of isotonic crystalloids within the first 3 hours.^(5)^ However, individualized assessment is critical; therefore, evaluation of fluid responsiveness through dynamic measures is recommended. Depending on the setting, performing a dynamic and individualized assessment of intravascular volume may not be feasible in the earliest phases of resuscitation. Beyond this phase, all efforts should be made to guide additional fluid administration by ongoing evaluations of volume status, fluid responsiveness, and tissue perfusion, and bedside ultrasound may be a valuable tool in this process. Fluid administration should be guided by dynamic assessments (e.g., passive leg raise, pulse pressure variation, or stroke volume response to a fluid challenge), which are superior to static measures. Limitations include arrhythmias, low tidal volumes, spontaneous breathing, and time constraints in resource-limited settings. In patients showing signs of fluid intolerance – such as worsening oxygenation or pulmonary congestion on lung ultrasound – further fluids should be avoided, even in the presence of fluid responsiveness.^(6)^ Serial bedside assessments, such as CRT, lactate clearance, central venous oxygen saturation (ScvO_2_), venous-arterial CO_2_ gap, and point-of-care ultrasound, support a goal-directed and physiologically coherent strategy. Crystalloids remain the first-line choice; balanced crystalloids, such as lactated Ringer's solution, are preferred over 0.9% saline given the current evidence of improvement in clinical outcomes, except in traumatic brain injury patients.^(5,7)^ A recent meta-analysis failed to show mortality improvement in using of albumin,^(8)^ however it may be considered in patients who have already received large volumes of crystalloids and in cirrhotic patients. Prolonged supplementation aimed at maintaining serum albumin levels ≥ 30g/L throughout the ICU stay, or up to day 28 may reduce early fluid balance; however, this strategy has not consistently translated into improved clinical outcomes, with at most a modest signal of benefit observed in the most severely ill patients.^(9)^ Once stabilized, fluid de-escalation and removal strategies should be implemented to avoid cumulative overload.

## 3. DO NOT FORGET TO USE VASOPRESSORS WISELY

Norepinephrine is the first-line vasopressor and should be started promptly – even through a peripheral line – in patients with life-threatening hypotension or persistent shock despite fluids. Vasopressors act through their adrenergic stimulation, promoting vasoconstriction and can also mobilize unstressed venous volume, thereby increasing preload. Nevertheless, catecholamines carry risks, including tachyarrhythmias, increased myocardial oxygen demand, peripheral and splanchnic ischemia, immune dysregulation, and endothelial injury.^(10)^

Optimal vasopressor use requires a multimodal strategy to spare catecholamines. Adequate fluid resuscitation should be ensured, and the target MAP should initially be maintained at 65 - 70mmHg, with later individualization based on comorbidities and patient-specific factors, as higher thresholds increase vasopressor exposure without proven benefit.^(5,11)^ Although a recent study reported disappointing effects of raising diastolic arterial pressure (DAP) on tissue perfusion,^(4)^ DAP-based metrics may still serve as complementary physiological signals when assessing vasopressor support. Patients with chronic arterial hypertension may warrant higher individualized MAP targets with careful evaluation of perfusion response; however, a recent meta-analysis of individual patients failed to demonstrate benefit in mortality with this strategy.^(12)^ Adults over 65 years old might benefit from lower targets (60 - 65mmHg).^(13)^ Low-dose hydrocortisone (200mg/day) may improve vascular responsiveness and facilitate vasopressor weaning,^(14)^ while vasopressin can be added as a second-line agent to reduce catecholamine requirements,^(15)^ although a definitive norepinephrine dose (base or salt) threshold has not yet been established. Avoidance of aggravating factors, such as acidosis or excessive sedation, is also critical for stabilization. Management should always be individualized according to patient characteristics and clinical response.

## 4. DO NOT FORGET TO ASSESS AND TREAT CARDIAC DYSFUNCTION

Septic cardiomyopathy, present in 40 - 70% of septic patients, is a reversible myocardial dysfunction typically resolving within 7 - 10 days.^(16)^ Its mechanisms include inflammatory injury, mitochondrial dysfunction, and myocardial edema. It may manifest as left ventricular systolic or diastolic dysfunction or right ventricular impairment. Bedside echocardiography may help recognize distinct phenotypes, from hyperdynamic dysfunction to right or left ventricular dysfunction, and guide individualized management. Ejection fraction assessment may be misleading due to preload and afterload changes, and cardiac biomarkers such as troponin, while often elevated, lack diagnostic specificity

Despite the high prevalence of septic cardiomyopathy, only a minority of patients ultimately require inotropic support, as most maintain adequate cardiac output with standard resuscitation and vasopressor therapy. Importantly, coronary perfusion pressure depends primarily on diastolic arterial pressure rather than MAP, which reinforces the need to avoid excessive vasodilation and to optimize vascular tone in selected cases.^(17)^

In patients with persistent signs of impaired perfusion suggestive of low cardiac output, dobutamine or epinephrine may be used to improve cardiac output, guided by mixed/central venous oxygen saturation, venous-arterial CO_2_ gap, CRT, or lactate clearance. Levosimendan is not recommended. To date, no therapy has robust evidence for improving survival in septic cardiomyopathy.^(5)^

## 5. DO NOT FORGET THE KEY POINT TO MANAGE REFRACTORY SEPTIC SHOCK

Recently, a Delphi consensus proposed a broad clinical definition of refractory septic shock, characterized by persistent hypoperfusion and the requirement for high doses of vasopressors in the absence of hypovolemia and after exclusion of alternative causes of shock using bedside ultrasound. Standardization of this definition is essential, as the lack of uniform criteria compromises comparability across studies and limits the development of consistent, evidence-based therapeutic protocols.^(18)^ The management requires a structured stepwise approach.^(19)^ A mandatory step is the reassessment of antimicrobial therapy, ensuring both appropriate spectrum and dosing, as well as effective source control. In parallel, alternative causes of shock – such as pulmonary embolism, pneumothorax, or hemorrhage – must be excluded, and patients should be reevaluated for septic cardiomyopathy.

Hemodynamic optimization includes revisiting fluid responsiveness. If vasopressin has not yet been initiated, it should be started at doses up to 0.04U/minute. Higher doses (up to 0.06U/minute) may be considered, although the supporting evidence is limited and potential adverse effects must be taken into account.^(20,21)^ When shock persists despite these interventions, escalation strategies such as the addition of epinephrine^(5)^ or angiotensin II^(22)^ may be required, although the latter remains limited in availability.

In cases of persistent vasoplegia, methylene blue can be considered.^(5)^ By inhibiting guanylate cyclase and nitric oxide synthase, it has the potential to reduce vasopressor requirements, although evidence of impact on mortality is still lacking.^(23)^

## CONCLUSION

Septic shock remains one of the most demanding conditions encountered in intensive care, requiring timely recognition and a systematic approach to management. Intensivists must not overlook the essential steps of identifying and treating hypoperfusion, ensuring appropriate fluid resuscitation, using vasopressors judiciously, recognizing and managing septic cardiomyopathy, and applying a structured strategy for refractory shock. Optimized intensive care unit management is essential to improve short-term outcomes and may also play a pivotal role in shaping long-term recovery, functional outcomes, and quality of life for survivors.

## Data Availability

No datasets were generated or analyzed during the current study.
